# The relationship between cigarette smoking and the tongue microbiome in an East Asian population

**DOI:** 10.1080/20002297.2020.1742527

**Published:** 2020-03-25

**Authors:** Noriaki Sato, Masanori Kakuta, Eiichiro Uchino, Takanori Hasegawa, Ryosuke Kojima, Wataru Kobayashi, Kaori Sawada, Yoshihiro Tamura, Itoyo Tokuda, Seiya Imoto, Shigeyuki Nakaji, Koichi Murashita, Motoko Yanagita, Yasushi Okuno

**Affiliations:** a Department of Biomedical Data Intelligence, Graduate School of Medicine, Kyoto University, Kyoto, Japan; b Department of Nephrology, Graduate School of Medicine, Kyoto University, Kyoto, Japan; c Human Genome Center, The Institute of Medical Science, the University of Tokyo, Tokyo, Japan; d Department of Medical Intelligent Systems, Graduate School of Medicine, Kyoto University, Kyoto, Japan; e Health Intelligence Center, the Institute of Medical Science, The University of Tokyo, Tokyo, Japan; f Department of Oral and Maxillofacial Surgery, Hirosaki University Graduate School of Medicine, Aomori, Japan; g Department of Social Medicine, Hirosaki University Graduate School of Medicine, Aomori, Japan; h Department of Oral Health Care, Hirosaki University Graduate School of Medicine, Aomori, Japan; i COI Research Initiatives Organization, Aomori, Japan

**Keywords:** Oral cavity, microbiome, cigarette smoking, tongue, East Asia

## Abstract

**Background**: The oral microbiome, which consists of various habitats, has been shown to be influenced by smoking. However, differences in the tongue microbiomes of current and former smokers, as well as their resultant functional consequences, have rarely been investigated in East Asian populations.

**Methods**: We used *16S rRNA* amplicon sequencing of tongue-coating samples obtained from East Asian subjects who were current, former, or never smokers to identify differences in their tongue microbiomes and related metagenomic functions. Two sets of participants from 2016 to 2017 (n = 657 and n = 187, respectively) were analyzed separately.

**Results**: We found significant differences between the overall microbiome compositions of current versus never smokers (p = 0.0015), but not between former versus never smokers (p = 0.43) based on the weighted UniFrac distance. Twenty-nine of 43 investigated genera showed significantly different expression levels in current versus never smokers. *Neisseria* and *Capnocytophaga* were less abundant, and *Streptococcus* and *Megasphaera* were more abundant in current smokers. Moreover, the abundances of metagenomic pathways, including those related to nitrate reduction and the tricarboxylic acid cycle, were significantly different between current and never smokers.

**Conclusions**: The tongue microbiomes and related metagenomic pathways of current smokers differ from those of never smokers among East Asians.

Cigarette smoke contains many toxicants that can affect the oral cavity environment, which is the first body part to come into direct contact with smoke. The oral microbiome is known to play an important role in many systemic diseases including diabetes mellitus, rheumatoid arthritis, and stroke [1–3]. Thus, changes to the oral microbiome that are induced by external factors, including cigarette smoke, are of great interest.

The relationship between the oral microbiome (which consists of several different habitats) and cigarette smoking has gained increasing attention. Wu et al. explored this relationship in a large American cohort by sampling expelled mouthwash [4] and showed that certain pathways related to anaerobic, aerobic, and xenobiotic metabolism were influenced by the smoking behavior. While studies that investigated the relationship between smoking and the tongue microbiome have been performed [5,6], the detailed differences in the metagenomic functionality of the tongue microbiome in East Asian populations due to smoking have scarcely been investigated. Moreover, research using the exact sequence variants-based approach, which was found to be superior in terms of reproducibility and resolution compared to the operational taxonomic units-based approach, has never been performed in the context of exploring the relationship between the tongue microbiome and smoking.

Oral microbiomes were reported to have ethnicity-specific signatures in another study [7], and genetic variations of the host were reported to influence oral microbiomes’ structure [8,9]. In addition, the predominant oral microbiota were reported to be acquired early and persist through life [10]. Therefore, the effect of cigarette smoking on the overall oral microbiome structure could vary depending on geographic or ethnicity background.

The aim of this study was to investigate whether differences exist in the tongue microbiomes of East Asians based on their smoking statuses using exact sequence variants-based *16S rRNA* amplicon sequencing. The metagenomic implications of differences in the microbiota were also examined.

## Materials and methods

### Participants

This cross-sectional study was conducted according to the principles expressed in the Declaration of Helsinki and was approved by the Ethics Committee on Human Research at Hirosaki University (approval number: 2016-028, 2017-026). From among participants in the Iwaki Health Promotion Project, we selected those who underwent tongue-coating analyses in 2016 (n = 1,139) and 2017 (n = 1,059); tongue coating refers to the normal mucosa present on the dorsal surface of the tongue. That project aimed to gather annual comprehensive clinical data from healthy individuals using over 2,000 questionnaires as well as collecting laboratory data, and has been ongoing since 2005 with the goal of attaining insights into the mechanisms of the onset of complex diseases. Written informed consent was obtained from all participants prior to initiating the study.

### Covariate assessment and participant selection

The participants’ covariate information was obtained from questionnaires administered upon induction into the study. Smoking status was classified as current, former, and never. Drinking status was classified as non-drinker, former drinker and current drinker. For current and former smokers, information on the ages at commencing and (when applicable) ceasing the smoking habit, as well as the number of cigarettes smoked per day, were also collected. Body mass index (BMI) was calculated based on height and weight of the participants. We also described the predicted percentage of forced expiratory volume in one second and pack-year index (the number of cigarettes smoked per day divided by 20, multiplied by the number of years of smoking) across the three groups. Pack-years index was reported to be related to the risk of lung cancer [11]. Oral health status was examined by dentists, and the natural tooth number, presence of dental caries and periodontal status were recorded. Dental caries was divided into two categories of either presence or absence. Periodontal status was classified to suspected of having periodontal disease or not, based on the findings of tartar, gum bleeding and gingival pocket depth. The study’s exclusion criteria were as follows: (1) Those who were younger than 20 years of age or were 90 years or older; (2) those who were prescribed oral antimicrobials or steroids on admission; (3) those with estimated glomerular filtration rate below 30 mL/min/1.73 m^2^ as calculated from serum creatinine levels and age at admission; (4) those who were on antihypertensive drugs, who reported having hypertension; (5) those whose hemoglobin A1c was 6.5% or above, who were taking oral hypoglycemic agents, or who reported having diabetes mellitus on their questionnaires; (6) those who had no teeth; (7) those who had prescribing records of probiotics; and (8) those with missing information regarding any of their covariates.

### Sample collection and microbiome assay

Tongue-coating samples were obtained via cotton swabs on the morning of admission before breakfast and oral brushing and were stored in a specimen tube containing 1 mL of 4 M guanidium thiocyanate, 100 mM Tris-HCl (pH 8.0), 40 mM EDTA and 0.001% bromothymol blue with the cotton swab inside. Samples were stored at 4°C until use. The detailed library preparation method, including PCR conditions, has been described previously [12]. Briefly, the samples were mixed with zirconia beads using a FastPrep 24 instrument (MP Biomedicals, Santa Ana, CA, USA). DNA was extracted from the bead-treated suspensions using an automatic nucleic acid extractor and MagDEA DNA 200 (GC) or MagDEA Dx SV (Precision System Science, Chiba, Japan). The *16S rRNA* gene amplicons covering the V3–V4 region were amplified using the universal primer sets described previously [12]. Sequencing was performed using a paired-end, 2 × 300-base pair cycle run on an Illumina MiSeq sequencing system. Quality control, trimming, merging, and chimera detection were performed using DADA2 (maxN = 0, maxEE = 1 for both forward and reverse reads, truncQ = 2) [13]. Bacterial taxonomy was assigned using the Ribosomal Database Project version 16 as a reference [14]. The number of total denoised reads included in the analysis was 19,392,711 sequences (mean ± standard deviation [SD]: 29,517 ± 9,356 sequences per sample; range, 14,526–142,475). All sequences were aligned by the computer program MUSCLE [15], and multiple sequence alignments were filtered by the program Noisy [16]. The phylogenetic tree was subsequently generated using FastTree [17]; the tree was rooted using the outgroup rooting method. We inferred the metagenomic function of the microbial community using the PICRUSt2 [18] algorithm, which used the following tools and algorithms internally: HMMER [19], EPA-NG [20], gappa [21], and castor [22]. The MetaCyc [23] pathway abundances were predicted from ‘Enzyme Commission’ numbers abundances by MinPath [24]. The weighted nearest sequenced taxon index (NSTI) values, which show the phylogenetic distance of the samples and the fully sequenced genome, were calculated for prediction reliability.

### Statistical analyses

Continuous variables were compared using one-way analysis of variance (ANOVA), while categorical variables were compared using the chi-squared test when assessing the participants’ background characteristics. The alpha diversity was measured by the observed species counts as well as Shannon’s and Simpson’s diversity indices based on the rarefied table for the sample with the minimum sequence count, and was compared across the three groups using a linear model. The overall microbiome differences were tested using permutational multivariate ANOVA based on the Bray–Curtis dissimilarity, the unweighted and weighted UniFrac distance [25] by the adonis function in the R package vegan. The unweighted, weighted UniFrac distances and Bray–Curtis dissimilarity were calculated based on log-transformed abundance with a pseudocount of 1. We performed a pairwise comparison of groups by assessing the beta diversity, whereupon the p-values were adjusted for multiple comparisons. The amplicon sequence variants that were present in less than 15% of the total dataset were excluded, and the remainder were then categorized into 9 phyla, 13 classes, 17 orders, 28 families, and 45 genera while excluding unassigned taxa. The Wald test was used to analyze the differential abundance of taxa and inferred pathways using DESeq2 [26], which estimates the log2 fold change between conditions using generalized linear models with a negative binomial distribution. For a differential abundance of pathway analysis, the rounded pathways’ abundances were tested for differences using DESeq2. Age was divided into six categories partitioned by 20, 30, 40, 50, and 65 years old. The number of natural teeth was divided into three categories partitioned by 1, 10 and 20. BMI was classified to four categories according to World Health Organization criteria when describing the participants’ background or incorporated into statistical models or tests. All models were adjusted for possible confounders; age, sex, BMI, drinking status, the number of teeth, presence of caries, and periodontal status. We performed the discovery study in the 2016 cohort and the validation study in the 2017 cohort. All statistical tests were two-sided, and a p-value or false discovery rate-adjusted p-value of less than 0.05 were considered statistically significant. All microbial and statistical analyses were conducted using R 3.5.0 and the R library phyloseq [27]. Figures were generated using the R library ggplot2 [28].

## Results

### Microbiome diversity

**Figure 1.**
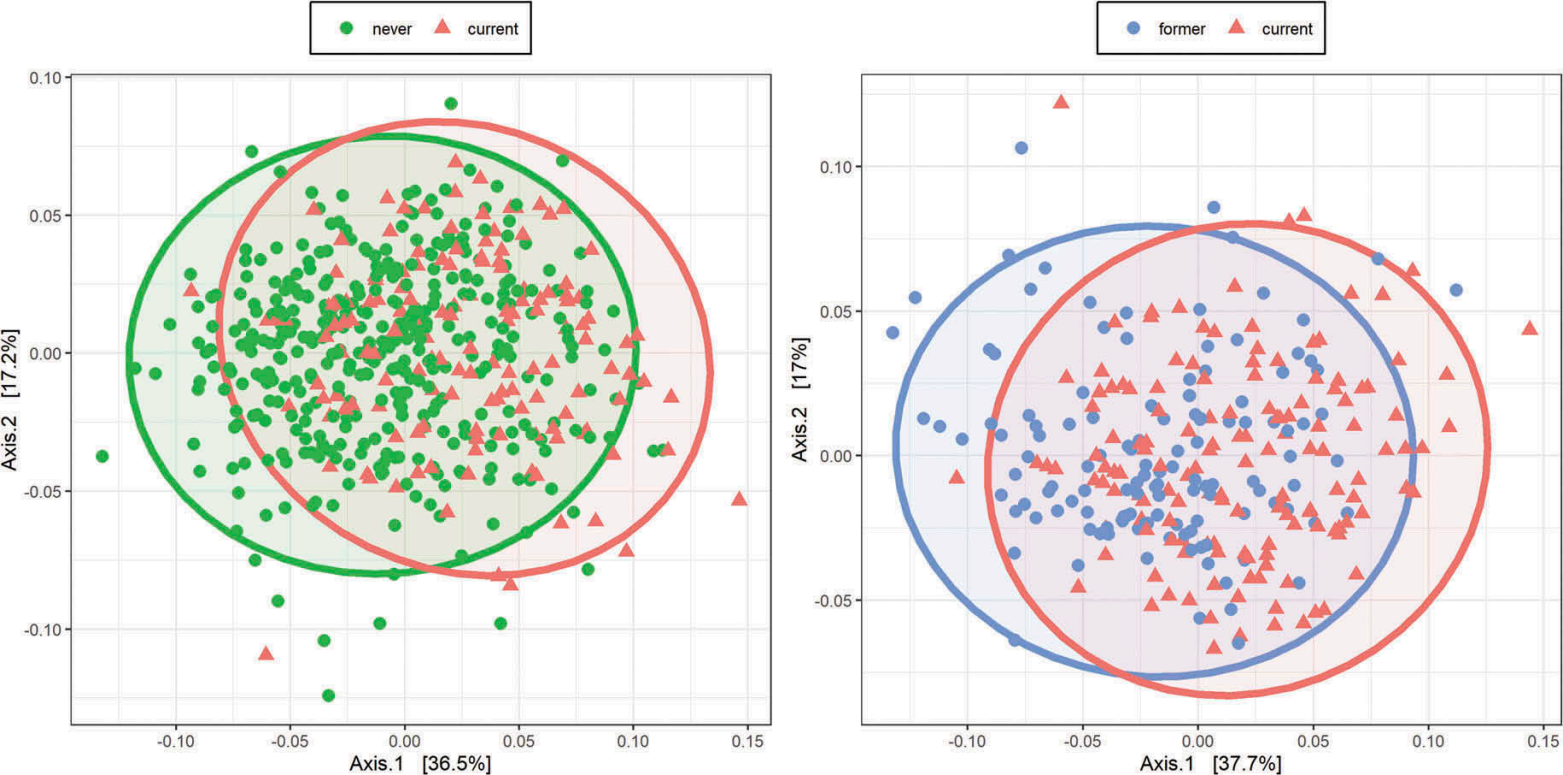
Overall microbiome composition

We first analyzed the data of 657 participants who were swabbed in 2016, the overall characteristics of whom are shown in Table 1. There were statistically significant differences between current and never smokers (pseudo-F = 25.65, R^2^ = 0.045, p = 0.0015 for the weighted UniFrac; Figure 1) and between current and former smokers (pseudo-F = 20.14, R^2^ = 0.068, p = 0.0015 for the weighted UniFrac; Figure 1). On the other hand, there was no statistically significant difference between former and never smokers (pseudo-F = 0.89, R^2^ = 0.0017, p = 0.43 for the weighted UniFrac). The results were similar when comparison was made in unweighted UniFrac distance and Bray–Curtis dissimilarity (Table S1).

**Table 1.** Overall participants’ background in 2016

Clinival values	Never smokers (n = 384)	Former smokers (n = 129)	Current smokers (n = 144)	p-value
Age (years), mean (SD)	49.78 (15.21)	48.03 (11.27)	43.99 (10.87)	<0.001
Sex: # female, %	283 (73.7)	60 (46.5)	51 (35.4)	<0.001
eGFR (mL/min/1.73 m^2^), mean (SD)	81.69 (14.78)	81.13 (13.23)	83.53 (12.77)	0.304
HbA1c (%), mean (SD)	5.67 (0.30)	5.66 (0.28)	5.65 (0.30)	0.742
Systolic blood pressure (mmHg), mean (SD)	120.78 (17.15)	120.49 (15.69)	116.60 (15.50)	0.031
Diastolic blood pressure (mmHg), mean (SD)	73.88 (11.62)	75.50 (11.95)	73.58 (12.94)	0.342
Pack-year index, mean (SD)	NaN (NA)	13.44 (15.52)	18.85 (12.36)	0.002
FEV1.0%, mean (SD)	82.73 (6.60)	81.72 (6.41)	81.35 (6.85)	0.063
BMI category # (%)				0.298
0–18.5 kg/m^2^	34 (8.9)	10 (7.8)	18 (12.5)	
≥18.5–25 kg/m^2^	275 (71.6)	100 (77.5)	95 (66.0)	
≥25–30 kg/m^2^	64 (16.7)	18 (14.0)	29 (20.1)	
≥30 kg/m^2^	11 (2.9)	1 (0.8)	2 (1.4)	
Number of teeth category # (%)				0.013
≥1–10	16 (4.2)	0 (0.0)	4 (2.8)	
≥10–20	38 (9.9)	8 (6.2)	5 (3.5)	
≥20	330 (85.9)	121 (93.8)	135 (93.8)	
Drinking status # (%)				<0.001
Non-drinker	253 (65.9)	25 (19.4)	53 (36.8)	
Former drinker	12 (3.1)	10 (7.8)	1 (0.7)	
Current drinker	119 (31.0)	94 (72.9)	90 (62.5)	
Caries present # (%)	115 (29.9)	37 (28.7)	66 (45.8)	0.001
Suspected of having periodontal diseases # (%)	271 (70.6)	94 (72.9)	122 (84.7)	0.004

Age, systolic and diastolic blood pressure, HbA1c, eGFR, FEV1.0%, and pack-year index were compared using one-way ANOVA, while number of teeth, sex, drinking status, presence of caries, periodontal status and BMI category were compared using the chi-squared test.

A principal coordinate analysis was performed on the weighted UniFrac distance. The x- and y-axes represent the first and second principal coordinates with the proportion of variance. The 95% confidence ellipse is shown for each group. There was no statistically significant difference between former and never smokers; however, statistically significant differences were observed when comparing current versus former (right) as well as current versus never smokers (left).

The observed counts of species showed no statistically significant differences between former and never smokers (beta = 14.16, standard error (SE) = 8.30, p = 0.091) and current and never smokers (beta = 9.44, SE = 9.07, p = 0.30). Furthermore, there were no statistically significant differences between former and never smokers according to either Shannon’s diversity index (beta = 0.13, SE = 0.098, p = 0.18) or Simpson’s diversity index (beta = 0.0059, SE = 0.011, p = 0.60). However, there were statistically significant differences between current and never smokers according to Shannon’s diversity index (beta = −0.23, SE = 0.11, p = 0.037) and Simpson’s index (beta = −0.034, SE = 0.012, p = 0.0067).

**Figure 2.**
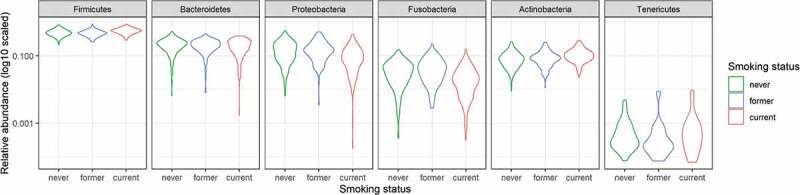
The violin plot representing the relative abundances of the phylum

**Figure 3.**
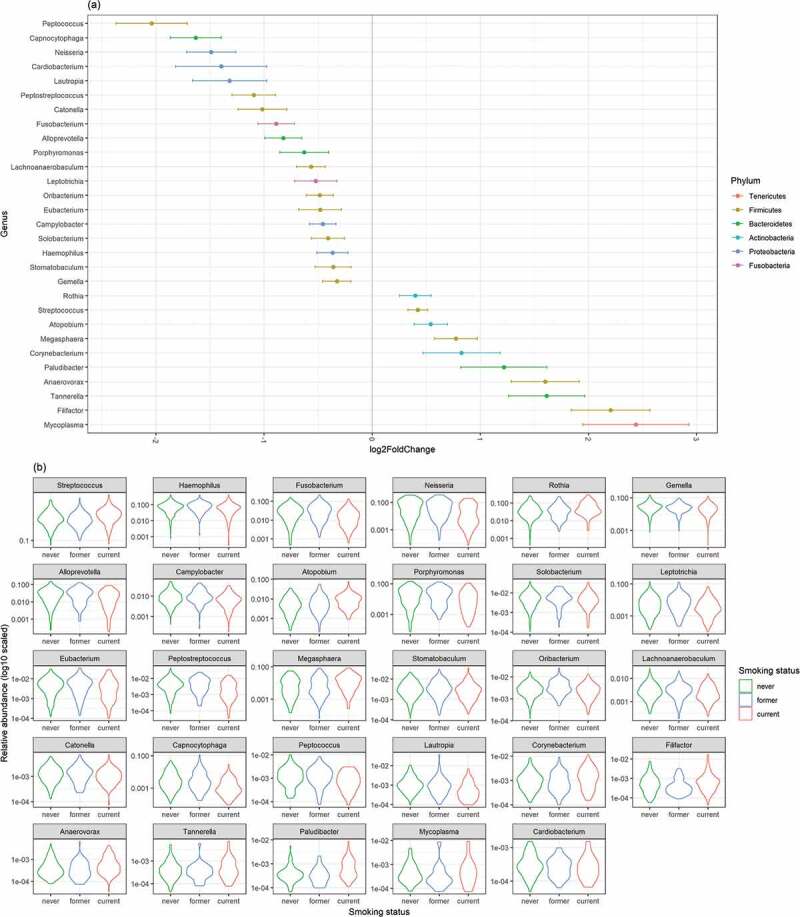
The result of comparison of bacterial abundance at the genus level

The abundance of each taxon was subsequently compared across groups using DESeq2. Six phyla showed significant differences between current and never smokers: *Actinobacteria* (p < 0.001) and *Firmicutes* (p < 0.001) were more abundant, while *Bacteroidetes* (p = 0.010), *Proteobacteria* (p < 0.001), and *Fusobacteria* (p < 0.001) were less abundant in current smokers (Table 2, Figure 2). Additionally, 12 classes showed significant differences between current and never smokers: *Bacteroidia, Betaproteobacteria*, and *Clostridia* (all p < 0.001) were less abundant in current smokers, while *Actinobacteria* (p < 0.001) and *Negativicutes* (p < 0.001) were more abundant in current smokers. Of the 43 genera, 29 showed significant differences between current and never smokers: *Streptococcus* (p < 0.001), *Megasphaera* (p < 0.001), *Anaerovorax* (p < 0.001), and *Atopobium* (p = 0.0021) were more abundant, while *Neisseria* (p < 0.001), *Capnocytophaga* (p < 0.001), and *Haemophilus* (p = 0.017) were less abundant in current smokers (Figure 3). There were no significant differences between former and never smokers. All ASVs presented in the 2016 cohort were summarized in Table S2. The results of differential genera abundance analysis and their representative sequences are summarized in Table S3.

**Table 2.** Results of differential abundance of phyla in 2016

Phylum	Base Mean	log2 FoldChange	lfcSE	Stat	pvalue	padj
Firmicutes	12084.30719	0.329331	0.052812	6.235921	4.49E-10	1.35E-09
Bacteroidetes	4805.37285	−0.22542	0.082985	−2.71639	0.0066	0.0099
Proteobacteria	3284.594671	−0.82262	0.110453	−7.44768	9.50E-14	4.27E-13
Fusobacteria	844.1792283	−0.69113	0.143631	−4.81187	1.50E-06	3.36E-06
Actinobacteria	2150.816342	0.651696	0.080319	8.113884	4.90E-16	4.41E-15
Tenericutes	4.223112698	3.648186	0.790434	4.615421	3.92E-06	7.06E-06

Base mean: the mean of normalized counts of all samples normalized for sequencing depth, lfcSE: standard error of log2 fold change, stat: Wald statistics, padj: adjusted p-value.

The x-axis shows the smoking status and y-axis shows the relative abundance (log10 scaled).

(a) The x-axis shows the shrinked log2 fold change of current smokers and standard error bars using never smokers as references, while the y-axis shows the genera. (b) The relative abundances are visualized by the violin plots. The x-axis shows the smoking status and y-axis shows the relative abundance (log10 scaled).

### Functional analysis

A metagenomic analysis was conducted to determine the functional consequences of different microbiota compositions using the PICRUSt2 algorithm. The mean ± SD of the overall weighted NSTI values was 0.063 ± 0.034. Among the 395 MetaCyc pathways predicted by the Enzyme Commission number abundance, 290 pathways existing in more than 15% of the participants were tested; 189 pathways were significantly different when comparing never smokers to current smokers. Among these pathways were those involved in denitrification, sulfate reduction, the tricarboxylic acid (TCA) cycle, glyoxylate cycle, aerobic respiration, 2-methylcitrate cycle, and several compound biosynthesis pathways such as ubiquinol, menaquinol, L-arginine or L-ornithine. One pathway (mycolyl-arabinogalactan-peptidoglycan complex biosynthesis) was significantly different between former and never smokers. The results of differential abundance analyses for pathways are summarized in Table S4.

### Validation study

**Figure 4.**
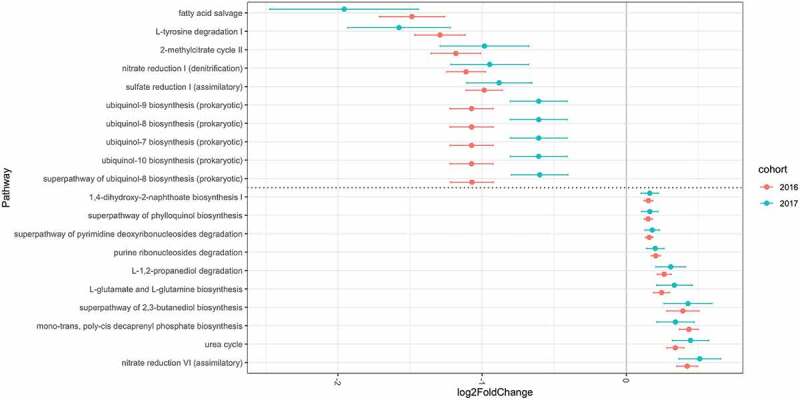
Log2 fold change in pathway abundance over never smoker levels

The results from samples obtained from subjects swabbed in 2016 were compared to those of 187 participants swabbed in 2017 who met the inclusion criteria; the latter group was independent of the former and served as a validation cohort. The number of total denoised reads included in the validation study was 4,407,131 sequences (mean ± SD: 23,568 ± 8,403 sequences per sample; range 10,136–56,680). The mean ± SD overall weighted NSTI values in the 2017 analysis were 0.100 ± 0.043. The analysis included 7 phyla, 11 classes, 14 orders, 25 families, and 38 genera. The overall characteristics of the participants included in the validation study are shown in Table S5. The overall microbial composition was not significantly different between former and never smokers (pseudo-F = 0.37, R^2^ = 0.0025, p = 0.96). However, a significant difference was observed between current and never smokers (pseudo-F = 4.85, R^2^ = 0.032, p = 0.006) and current and former smokers (pseudo-F = 3.23, R^2^ = 0.037, p = 0.017). When abundance on the genus level was compared using DESeq2, 10 of the 38 genera showed significant differences between current and never smokers: *Atopobium* (p = 0.016) and *Megasphaera* (p = 0.020) were more abundant, while *Peptostreptococcus* (p = 0.001) and *Capnocytophaga* (p < 0.001) were less abundant in current smokers. None of the genera showed significant differences between former and never smokers. Metagenomic analysis showed that, among the 284 MetaCyc pathways present in more than 15% of the participants, 82 showed significant differences between current and never smokers. There were no significant differences between former and never smokers in terms of the pathway abundance. All ASVs presented in the 2017 cohort were summarized in Table S6. The results of differential abundances in the genera and of pathways in the validation analysis are summarized in Tables S7 and S8. The common significant results of pathways for the two cohorts were shown in Table S9. There were 75 pathways both significant in the 2016 and 2017 cohorts, and 70 pathways were changed to the same direction. Among these, the pathways with highest and lowest 10 log2 fold change in the 2016 cohort are shown in Figure 4 along with log2 fold change in the 2017 cohort.

The x-axis shows the log2 fold change and standard error bars using never smokers as references, while the y-axis shows the selected pathways. The pathway above dotted line indicates these pathways were less abundant in current smokers, while the pathway below dotted line indicates more abundant in current smokers.

## Discussion

Our investigation of the relationship between the tongue microbiome composition and smoking status revealed that the tongue microbiomes and related metagenomic pathways of current smokers differed from those of never smokers, while no significant differences were found between the tongue microbiomes of former and never smokers. Some of these results reproduced when repeated with a different year’s cohort. Our study is valuable in that it investigated subjects specifically from an East Asian population who were relatively younger in mean age than those in previous studies; moreover, newly developed algorithms were used to profile the bacteria and determine their functionality.

There was a significant difference in the tongue microbiome composition of current and never smokers. The alpha diversity was lower in current smokers than in never smokers, with a significantly different Simpson’s index and Shannon’s index. These data indicated differences in evenness and richness between current and never smokers. Beta diversity differed significantly in both analyses. Our findings of several differences in the genera and pathways between these groups are consistent with those of previous studies conducted by Wu et al. (which sampled ejected mouthwash) and Mason et al. (which sampled subgingival plaque), where genera such as *Neisseria* and *Capnocytophaga* were less abundant in current smokers than in never smokers while *Atopobium* and *Megasphaera* were more abundant [4,29]. Despite such similarities, however, there have been too few studies investigating the relationship between tongue coating and smoking; moreover, the results of these studies are not directly comparable because the microbial composition of the oral cavity varies by anatomical site [30]. The tongue and salivary microbiomes are reported to be highly similar, although differences exist with a small effect size [31]. Another study that examined the microbiome of the tongue and other oral/nasal sites as a function of smoking status found no differences in microbiome composition [6].

The commensal bacterial genus *Neisseria* is consistently found to be less abundant in current smokers in most studies investigating the relationship between smoking and oral microbiome. A smoking habit creates an anaerobic environment in the oral cavity, which favors anaerobic bacteria such as *Atopobium* over aerobic bacteria such as *Neisseria* [32]. Some species belonging to the *Neisseria* and *Streptococcus* genera were reported to grow more rapidly on natural teeth than on dentures [33]. Another study revealed a relationship between the condition of the oral environment, such as the number of natural teeth and percentage of carried teeth, and the tongue microbiome composition [34]. In our current study, we performed the analysis controlling for oral health status.

The oral microbiome plays a key role in metabolism and degradation. Our study specifically investigated the functionality of the tongue microbiome using PICRUSt2, by determining the involved MetaCyc pathways. PICRUSt2 was reported to be more accurate than other tools [18], and we utilized MinPath to infer the functionality of the microbiome, which is more stringent than the naïve mapping approach that was used in other studies, thus reducing the rate of false-positive results. Metagenomic content analysis revealed that pathways that differed between groups included those involving denitrification or sulfate reduction. Moreover, some aerobic respiration pathways such as the TCA cycle were found to be less active in current smokers, which is expected considering that aerobic bacteria were less abundant in current smokers. Moreover, anaerobic bacteria were more present in current smokers, and anaerobic fermentation pathways like pyruvate fermentation to acetate and lactate were more abundant in current smokers. Additionally, the 2-methylcitrate cycle, which is a pathway that metabolizes toxic propionyl-CoA into pyruvate, was less abundant in current smokers [35]. These pathway changes included some key metabolic pathways such as the TCA cycle and urea cycle, which were confirmed in the validation study. A previous study showed that the oral microbiomes and their associated pathways in former and never smokers were generally similar, and our study confirmed this to also be the case in the tongue microbiome, as there were no significant differences in taxonomic and pathway comparison. Moreover, smoking-related tongue microbiome changes appear not to be permanent [4].

The relationships between diseases and oral microbiome were previously investigated in several studies. One showed that patients with periodontitis had less abundant *Neisseria* in the oral mucosa [36], and another showed that *Capnocytophaga* and *Veillonella* were significantly more abundant in patients with lung cancer, and could, therefore, be potential biomarkers for this disease [37]. Moreover, children with autism spectrum disorder have less abundant *Prevotella* and *Porphyromonas* but more abundant *Streptococcus* in their oral microbiomes according to a recent analysis [38]. In the oral microbiomes of patients with colorectal cancer, genera such as *Neisseria, Prevotella, Haemophilus*, and *Streptococcus* were found to be less abundant than in controls [39]. The abundance of some of these genera whose quantities were potentially associated with diseases differed between current and never smokers in the tongue microbiome, though the natures of these associations were not confirmed in the setting of the present study.

The strength of our study was a relatively large sample size and the fact that we adjusted for possible confounding variables. Moreover, we used the exact sequence variant-based method, which is considered superior to operational taxonomic units-based analysis in terms of improved resolution and reproducibility [40]. Additionally, compared to Wu et al.’s cohort, ours comprised a relatively younger population in mean age; this indicated that the previously reported differences between the oral microbiomes of current and never smokers were also likely to be true in a younger population [4].

Our study also had limitations. First, prescription information (used for covariate adjustment) was based on the subjects’ medication notebooks that were checked on admission; therefore, the duration of administration was unknown. Furthermore, information regarding smoking history was based on the participants’ recollection, which may have resulted in recall and reporting bias. Moreover, it was not possible to determine if differences in genera or pathways were directly related to the onset or pathogenesis of various diseases or health consequences, and longitudinal data for causal inferences were lacking. The discrepancies between the results we obtained for the 2016 and 2017 cohorts were presumed to be largely due to the difference in sample size. We did not sequence negative samples; therefore, the possibility of contamination cannot be excluded. Prebiotics and synbiotics information were lacking. Lastly, there are various methods to compare the differential abundance of compositional data, which were benchmarked in some papers; we only used the DESeq2 in our current study [41,42].

In summary, we found differences in tongue-coating microbiomes between never and current smokers in a cohort of East Asians, which was consistent with results from other studies that investigated different oral microbiome sites. The present study cannot provide clear evidence of any direct associations between changes in the microbiome due to smoking and specific diseases, however, several pathways related to important metabolic processes are influenced by smoking. Additional studies are required to investigate the effects of these differences on health.

## Supplementary Material

Supplemental Material

## Data Availability

The data that support the findings of this study are available from the corresponding author, Y.O., upon reasonable request.
